# Selection and validation of reference genes for measuring gene expression in *Toona ciliata* under different experimental conditions by quantitative real-time PCR analysis

**DOI:** 10.1186/s12870-020-02670-3

**Published:** 2020-10-01

**Authors:** Huiyun Song, Wenmai Mao, Zhihao Duan, Qingmin Que, Wei Zhou, Xiaoyang Chen, Pei Li

**Affiliations:** 1 Guangdong Key Laboratory for Innovative Development and Utilization of Forest Plant Germplasm, Guangzhou, 510642 China; 2 State Key Laboratory for Conservation and Utilization of Subtropical Agro-bioresources, Guangzhou, 510642 China; 3 South China Agricultural University, College of Forestry and Landscape Architecture, Guangzhou, 510642 China

**Keywords:** *Toona ciliata*, RT-qPCR, Reference gene, MeJA, *Hypsipyla robusta*, *TcMYB3*

## Abstract

**Background:**

Before studying gene expression of different organisms, it is important to determine the best reference gene. At present, the most accurate method of detecting gene expression is quantitative real-time PCR (RT-qPCR). With this method, reference genes that are stable in different biological systems and under different conditions can be obtained. *Toona ciliata* Roem (*T. ciliata*). is a valuable and fast-growing timber specie. In this study, 20 reference genes were identified using RT-qPCR, as a primary prerequisite for future gene expression analysis. Four different methods, geNorm, NormFinder, BestKeeper, and RankAggreg were used to evaluate the expression stability of the 20 candidate reference genes in various tissues under different conditions.

**Results:**

The experimental results showed that *TUB-α* was the most stably expressed reference gene across all samples and *UBC17* was the most stable in leaves and young stems under *Hypsipyla robusta* (*H. robusta*) and methyl jasmonate (MeJA) treatments. In addition, *PP2C59* and *UBC5B* were the best-performing genes in leaves under *H. robusta* treatment, while *HIS1* and *ACT7* were the best reference genes in young stems. The two best reference genes were *60S-18* and *TUB-α* after treatment at 4 °C. The expression of *HIS6* and *MUB1* was the most stable under PEG6000 treatment. The accuracy of the selected reference genes was verified using the transcription factor MYB3 (*TcMYB3)* gene.

**Conclusions:**

This is the first report to verify the best reference genes for normalizing gene expression in *T. ciliata* under different conditions, which will facilitate future elucidation of gene regulations in this species.

## Background

*Toona ciliata* Roem. belongs to the Meliaceae family, which is widely distributed in China, Australia, and India. Because of its straight trunks and russet wood, *T. ciliata* has the title of “Chinese Mahogany” [1]. But its population has declines sharply in the past century due to environmental degradation and destruction by humans, and it has been listed as one of the “National Class II Key Protected Endangered Plants” in China. *T. ciliata* has great economic value, for example, its wood is often used to produce high-end furniture, instruments and crafts [2]. More importantly, it is also a medicinal plant as a result of the rich chemical substances in its roots, stems and leaves [3]. Compounds that have been isolated from *T. ciliata* include ketones, steroids, and coumarins, many of which have antifungal, anti-glycation, or anti-tumor activities [4–7], and its flower extract has a therapeutic effect on gastric ulcers [8]. However, the yield of compounds isolated from *T. ciliata* is low. In addition, in previous research, it has been found that *T. ciliata* is very susceptible to the moth pest *Hypsipyla robusta* Moore [9] that eats mainly the young stems and causes the hollow branches to fail to grow and die in some cases. This pest is not only a regional issue in China, but also a worldwide problem. In some of the main areas where *H. robusta* is distributed, such as Australia and Brazil, *T. ciliata* also faces serious damage from *H. robusta* [10–12]. At present, there are no chemical or physical methods to prevent or control *H. robusta* effectively, and current pest control methods are time- and labor- consuming, thus not applicable to large-scale forest plantations [13]. It may help to pest control by obtaining insect-resistant plants through molecular breeding. In order to synthesize a desired compound related to the resistance mechanism, it is necessary to first explore the pathway and its related regulatory genes [14, 15]. Gene expression analysis is one of the most powerful tools to explore biosynthetic and insect-resistance mechanisms in *T. ciliata*. So far, the knowledge base ICG (http://icg.big.ac.cn) has collected reference genes from more than 120 plant species including *Arabidopsis* [16], peanut [17], cucumber [18], and soybean [19], except *T. ciliata.* Nor are there any literature references about the housekeeping genes in *T. ciliata,* which can be used for the standardization of gene expression.

RT-qPCR has good repeatability, high sensitivity, accurate quantification, and fast reaction, making it a powerful tool to carry out the entire PCR process and the most commonly used method of determining gene expression levels [20]. However, RT-qPCR can be affected by multiple sources of error, such as the amount of starting materials, the integrity of the RNA, and the efficiency of the enzymatic reactions. It is therefore necessary to introduce a stably expressed housekeeping gene as a reference for correction and standardization, so as to control the unnecessary errors generated within and between samples [21].

The commonly used housekeeping genes are those consistently express under all conditions, such as genes encoding actin (*ACT*), glyceraldehyde-3 phosphate dehydrogenase (*GAPDH*), and tubulin (*TUB*) [22]. However, more and more studies are now questioning the existence of genes that are stably expressed across different tissues, different experimental conditions, and different species. In order to ensure the accuracy of an experiment, it is important to select those suitable reference genes for specific experimental conditions [23]. Software packages, including geNorm [24], NormFinder [25], and BestKeeper [26], are widely used to assess the expression stability of candidate reference genes and determine the best choices. Many researchers have used these algorithms to successfully identify reference genes in different species [27, 28]. The use of reference genes in expression analysis has greatly facilitated research in plant development and evolutionary mechanisms in species where a reference genome sequence is available [29].

In this study, 20 candidate genes from *T. ciliata* transcriptome database generated by our group were investigated to determine the most suitable *T. ciliata* candidate gene(s) as the reference(s) for gene expression analysis using RT-qPCR technique under specific conditions including different tissues (mature leaves, young leaves, flowers, shoots and young stems) and treatments (4 °C, MeJA, PEG6000 and *H. robusta*), including actin 7 (*ACT7*), phosphoglycerate kinase (*PGK*), *60S* ribosomal protein L13 (*60S-13*) and L18 (*60S-18*), histone deacetylase 1 (*HIS1*) and 6 (*HIS6*), protein phosphatase 2 C57 (*PP2C57*) and C59 (*PP2C59*), ubiquitin-conjugating enzyme E2 5B (*UBC5B*) and 17 (*UBC17*), S-adenosylmethionine decarboxylase proenzyme (*SAMDC*), elongation factor 1 (*EF1*) and 2 (*EF2*), peptidyl-prolyl cis-trans isomerase CYP95 (*PPIA*95) and CYP26–2 (*PPIA*26), *18S* rRNA (*18S*), tubulin alpha-3 chain (*TUB-α*), tubulin beta-5 chain (*TUB-β*), membrane-anchored ubiquitin-fold protein 1 (*MUB1*), and TIP41-like protein (*TIP41*). In addition, the *TcMYB3* gene was used to confirm the reliability and validity of the reference genes screened. MYB proteins, which constitute one of the largest family of transcription factors in plants, play important roles in plant growth and development, biotic and abiotic stress responses, and circadian rhythm regulation [30, 31]. For example, the R2R3 MYB transcription factor MdMYB30 modulates plant resistance against pathogens, and *Arabidopsis* transcription factor MYB102 increases plant susceptibility to aphids [32, 33]. Our research provided the best reference genes for RT-qPCR analysis of *T. ciliata* under different conditions, laying a foundation for studying molecular mechanisms in *T. ciliata* through gene expression analysis.

## Results

### Primer specificity, amplification efficiency, and expression profiles of candidate reference genes

Reverse-transcribed cDNA from each sample was used as a template with primers for standard PCR amplification. Electrophoresis verified all PCR products were specific with single bands in the gel (Fig. S1). The melting profiles of all amplified candidate reference genes using RT-qPCR showed single peaks (Fig. S2). A standard curve for each candidate was obtained by serial dilution, and their linear correlation coefficients were all greater than 0.99 (*R*^*2*^ > 0.99). The amplification efficiency for the 20 candidate reference genes ranged from 90.41% for *PPIA*95 to 102.44% for *PGK*. Further details of primers are given in Table 1.

**Table 1:** Candidate reference genes, primer sequences, and characteristics of PCR amplification in *T. ciliata*

Gene symbol	Gene Name	Primer: Forward/reverse	Amplification product size (bp)	standard curve	En	R^**2**^
*ACT7*	Actin-7	F: TGATTGGGATGGAAGCAGCA R: GAACATGGTTGAACCGCCAC	122	y = − 3.5133x + 29.711	0.9259	0.9931
*PGK*	Phosphoglycerate kinase	F: CCGCAAGCTTCTTTGCGATT R: GGCTTGGATATTGGACCCGA	145	y = − 3.2649x + 26.93	1.0244	0.9985
*60S-18*	60S ribosomal protein L18a-1	F: GCCTGGATGCCTTGTATGTTG R: GGGAAAGCACCAAGCAGTTTC	108	y = −3.5672x + 27.862	0.9332	0.9993
*60S-13*	60S ribosomal protein L13–1	F: CCAACATGGCACTCATTCGC R: TTCCCAAGATGTGCTCGCAA	200	y = −3.4076x + 29.173	0.9654	0.9929
*HIS6*	Histone deacetylase 6	F: ATTGTCCGGTGATAGGTTGGG R: GTCTCGTAGCACCAACAACG	153	y = −3.4932x + 29.484	0.9332	0.9965
*HIS1*	Histone acetyltransferase MCC1	F: CTGCACGAATTGTGCTGGTC R: ACTGCACGACATGTTGGGAT	193	y = −3.5229x + 30.411	0.9225	0.9959
*PP2C57*	Protein phosphatase 2C 57	F: TGTTGCAGCTTTACAAGGCG R: TGAACAAATCACCGCCTCCA	185	y = −3.3057x + 32.193	1.0068	0.9949
*PP2C59*	Probable protein phosphatase 2C 59	F: TAAGCGATCGCCAACAAGGA R: CACGAGCTGCTGAGTATGTGA	194	y = −3.3119x + 27.157	1.0042	0.9974
*UBC5B*	Ubiquitin-conjugating enzyme E2 5B	F: GGAGGACCCATGATTGTTGC R: TCGAAGCGGATCTTGAAGGAG	116	y = −3.3156x + 25.529	1.0026	0.9987
*UBC17*	Ubiquitin-conjugating enzyme E2–17 kDa	F: GCGTCGAAACGCATCTTGAA R: GAAACACCCCTCCCGCATAA	148	y = −3.4489x + 26.966	0.9496	0.999
*EF1*	elongation factor 1-alpha-like	F: CCGACCTTCTTCAGGTAGGAA R: TCCAAGGATGGTCAGACTCG	164	y = −3.4295x + 23.45	0.9570	0.9915
*EF2*	elongation factor 1-alpha-like	F: CACCCTTGGTGTGAAGCAAA R: GGTTGGTGGACCTCTCAATCA	200	y = −3.4128x + 27.077	0.9561	0.9992
*PPIA26*	Peptidyl-prolyl cis-trans isomerase CYP26–2	F: GAAGCTGAAGTTGGTTGCCC R: GACGACCAGGGCTGAAACAT	147	y = −3.4393x + 30.33	0.9532	0.9952
*PPIA95*	Peptidyl-prolyl cis-trans isomerase CYP95	F: ACCCGGCCTCTTATCTATGC R: ACAAGCTCCCCGAATACCAC	117	y = −3.4295x + 23.45	0.9041	0.9915
*SAMDC*	S-adenosylmethionine decarboxylase proenzyme	F: AGCGATCTGCTATGACCCTG R: CCCGCAGAACCTGATTGGTC	102	y = −3.3179x + 29.683	1.0017	0.9994
*18S*	18S rRNA factor 2	F: GCTGCTAAGAGAGAGCGGG R: GGGAGCTCAGAATGGGTTCG	128	y = −3.4317x + 30.095	0.9561	0.9987
*TUB-α*	Tubulin alpha-3 chain	F: TACAACAGTTGGCGGCTGAT R: TGTACCGCGGAGATGTTGTT	137	y = −3.3568x + 29.206	0.9857	0.9998
*TUB-β*	Tubulin beta-5 chain	F: ACACACGCTGGACTTGACAT R: TCGCTACCTAACTGCTTCGG	139	y = −3.3349x + 32.62	0.9946	0.9996
*MUB1*	Membrane-anchored ubiquitin-fold protein 1	F: GCATTCTTGCTCAATGGCCT R: GGTTGTAACTCCACCAGGGA	152	y = −3.3956x + 28.572	0.9701	0.9984
*TIP41*	TIP41-like protein	F: TGGTTGGAAGCAGGAAGGTT R: TTCACTTCCGCAGTATGGTG	133	y = −3.3332x + 31.127	0.9953	0.9992
*MYB3*	Transcription factor MYB3	F: CGCACCCATAACAACTCCCA R: TCTTTCACTTACTCCCTCTTCAGC	178	y = −3.4246x + 32.543	0.9589	0.9968

**Fig. 1:**
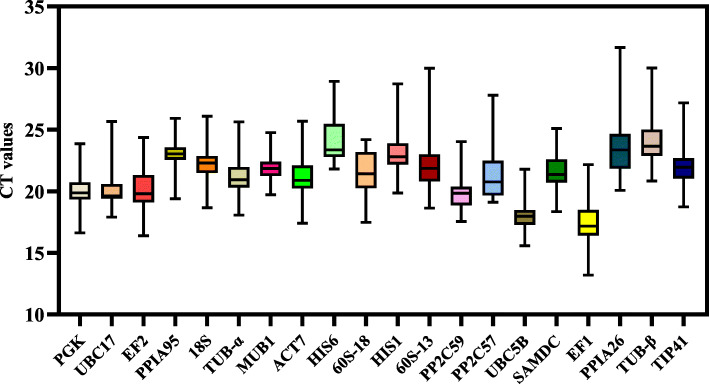
Distribution of threshold cycle (CT) values for 20 candidate reference genes across all samples. The middle line within each box represents the 50th percentile. The lower boundary and upper boundary of each box represent the 25th and 75th percentile respectively

The expression levels of all candidate reference genes were determined by RT-qPCR under all of the following conditions: different tissues, *H. robusta* treatment, 4 °C treatment, MeJA treatment, and PEG6000 treatment. The expression levels of candidate genes were very different across the samples. The maximum cycle threshold (CT) value was 31.66, and the minimum was 13.18 (Fig. 1). Among them, *PPIA*26 showed the highest expression abundance, with the maximum, minimum, and median of the CT values being 31.66, 20.07, and 23.36, respectively. *EF1* showed the lowest expression abundance, with the maximum, minimum, and median CT values being 22.16, 13.18, and 17.17, respectively. In addition, candidate genes exhibited significant variability in expression. *MUB1* and *UBC5B* had a relatively narrow range of CT values compared with other genes, indicating that they are more stably expressed. Notably, these results show that none of the genes are expressed stably across all conditions, so it is necessary to screen reference genes for *T. ciliata* under specific conditions.

### Stability of expression of candidate reference genes

The software packages geNorm, NormFinder, and BestKeeper were used to evaluate the expression stability of the 20 candidate reference genes under different experimental conditions. The R software RankAggreg package was used for overall ranking [34].

### GeNorm analysis

**Fig. 2:**
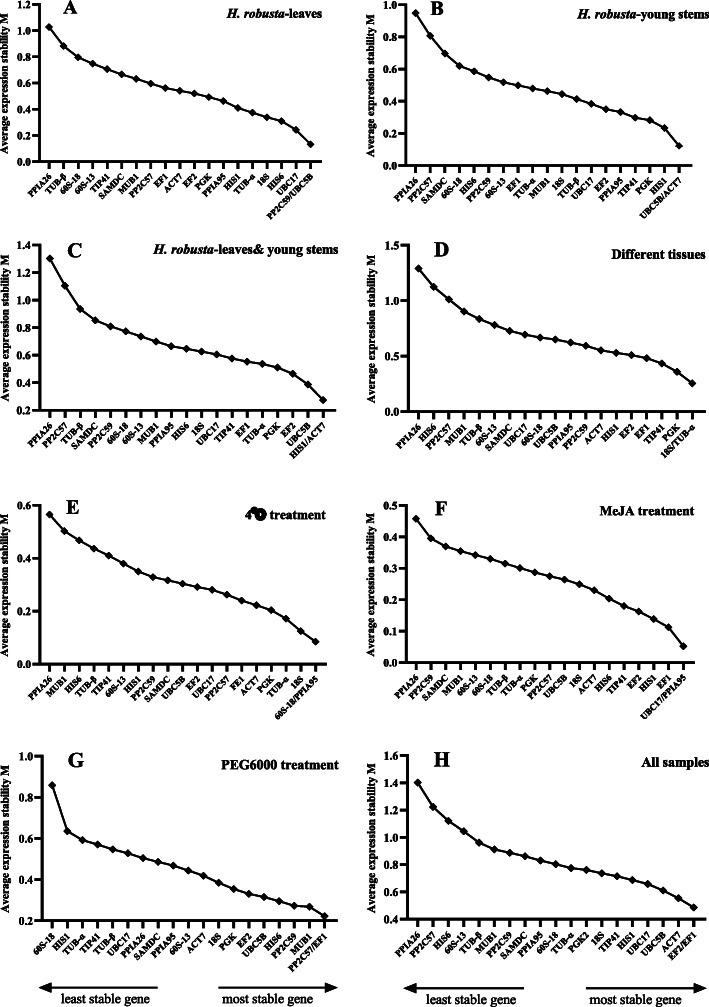
Average expression stability values (M) for 20 candidate reference genes calculated by geNorm

**Fig. 3:**
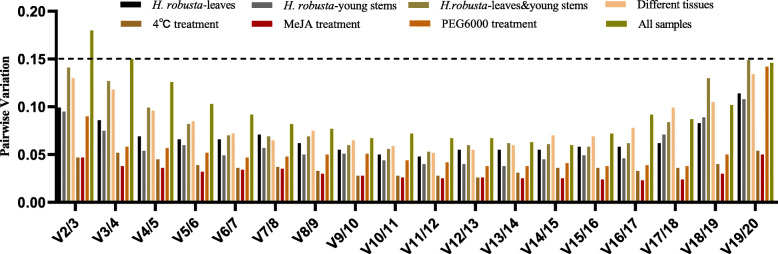
Pairwise variations (V) for the 20 candidate reference genes calculated by geNorm to determine the optimal number of reference genes for accurate normalization. The threshold used was 0.15

In geNorm analysis, M value is calculated for each pair of genes. The stability of gene expression is evaluated based on the M value; the genes with threshold M value below 1.5 are considered as stably expressed, and the gene with the lowest M value is regarded as the most stably expressed reference gene. The results of geNorm analysis of 20 candidate reference genes under different conditions are shown in Fig. 2a-h. The M values of all candidate genes from all the tested samples were below 1.5 (Fig. 3). Under *H. robusta* treatment, *UBC17*, *PP2C59*, and *UBC5B* were most stably expressed in leaves (Fig. 2a), while *HIS1*, *UBC5B*, and *ACT7* exhibited few expression fluctuations in young stems (Fig. 2b). Data Analyses from the two tissues under *H. robusta* treatment showed that *UBC5B*, *HIS1*, and *ACT7* were with the most stable expression as their M values are the lowest (Fig. 2c). The most stably expressed genes across different tissues were *18S* and *TUB-α*, with M values around 0.2 (Fig. 2d). *PPIA*95 showed good stability under both 4 °C and MeJA treatments; *60S-18* and *UBC17* were stably expressed only under 4 °C and MeJA treatment, respectively (Fig. 2e and f). The two genes with the lowest M values under drought stress which was simulated by PEG6000 treatment were *PP2C57* and *EF1* (Fig. 2g). And *EF2* and *EF1* had the highest stability with M value of 0.49 (Fig. 2h).

In general, it is more reliable to use multiple reference genes are more reliable than a single reference gene for quantitative gene analysis. Given this, geNorm calculates the pairwise variation (V_n/n + 1_) of the normalization factor after the introduction of a new reference gene, and determines the optimal number of reference genes based on this ratio. The default V_n/n + 1_ value for the software is 0.15. If the ratio is less than 0.15, the number of internal gene combinations that can meet the requirements for relative quantification is n, otherwise another reference gene needs to be introduced. In our study, the values of pairwise variation V_2/3_ under conditions with *H. robusta* treatment, 4 °C treatment, MeJA treatment, PEG6000 treatment, and different tissues, were all less than 0.15, indicating that the optimal number of reference gene combinations is two (Fig. 3). Across all samples, pairwise variation (V_2/3_) was 0.180, V_3/4_ was 0.15, and V_4/5_ was 0.126, indicating that the addition of the third and fourth reference genes has different impacts on the results. It always better to use fewer reference gene due to the time and cost economy consideration, hence the best reference gene combination was *EF2*, *EF1*, *ACT7* and *UBC5B* for all the samples.

### NormFinder analysis

**Fig. 4:**
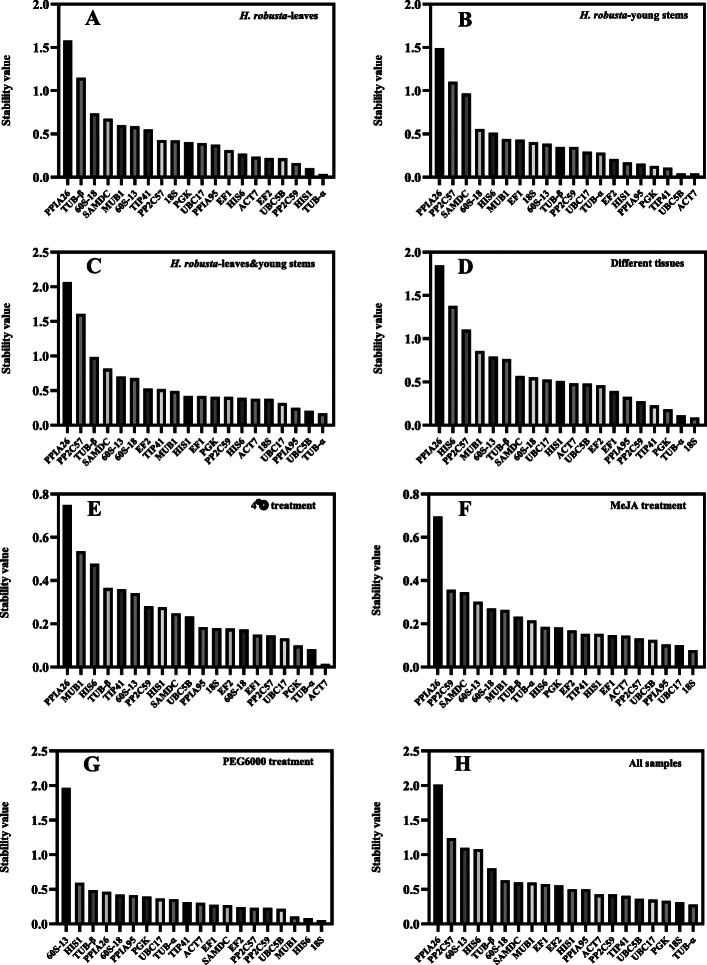
Expression stability of the 20 candidate reference genes as calculated by NormFinder

In order to further determine the stability of candidate reference genes, NormFinder was used to re-analyze the data. The results are shown in Fig. 4a-h. Under *H. robusta* treatment, the top three genes with stable expression in leaves were *TUB-α* (stability value =0.038), *HIS1* (0.105), and *PP2C59* (0.161) (Fig. 4a), while the most stable genes were *ACT7* (0.042), *UBC5B* (0.042), and *TIP41* (0.109) in young stems (Fig. 4b). The top three reference candidates in two tissues (leaves and stems) were *TUB-α* (0.170), *UBC5B* (0.206), and *PPIA*95 (0.250) (Fig. 4c). The genes *18S* (0.088) and *TUB-α* (0.112) had lower stability values across different tissues since they showed the most stable expression, which is consistent with the results of geNorm analysis (Fig. 4d). However, *ACT7* (0.014), *TUB-α* (0.082), and *PGK* (0.099) were the most stable candidate genes under 4 °C treatment (Fig. 4e), which is inconsistent with the results of geNorm analysis; this may be due to the fact that the two software packages use different algorithms. Under MeJA treatment, the two most stable reference genes were *18S* (0.078) and *UBC17* (0.100) (Fig. 4f), while under PEG6000 treatment, the two most stable were *18S* (0.055) and *HIS6* (0.082) (Fig. 4g). *TUB-α* (0.281), *18S* (0.316), and *PGK* (0.335) were the three genes with the lowest stability values for all samples (Fig. 4h). It is not consistent with geNorm analysis which showed that PPIA26, PP2C59 and HIS6 were the most unstable genes.

### BestKeeper analysis

BestKeeper takes as input the CT data for each gene-primer pair combination and calculates the coefficient of variation (CV) and standard deviation (SD), as shown in Table 2. The stability of genes is evaluated by the value of CV ± SD. More stable genes have a lower value of CV ± SD. *UBC17* (1.16 ± 0.23) and *18S* (1.62 ± 0.36) were the most stable genes in leaves under *H. robusta* treatment, and the expression of *HIS1* (1.40 ± 0.31) and *UBC17* (1.70 ± 0.33) were the most stable in young stems. Genes with the most stable expression across two tissues were *UBC17* (1.73 ± 0.34) and *18S* (2.12 ± 0.47), as was the case in leaves. *HIS6* (5.41 ± 1.38) and *MUB1* (5.48 ± 1.16) were the most stably expressed genes in the different tissues, *18S* (0.73 ± 0.16) and *60S-18* (0.83 ± 0.18) in 4 °C treatment, *60S-18* (0.67 ± 0.14) and *EF1* (0.83 ± 0.14) in MeJA treatment, *PPIA*26 (3.89 ± 0.87) and *60S-18* (4.48 ± 1.02) in PEG6000 treatment. For all samples, *MUB1* (3.95 ± 0.87) showed the highest value for expression stability.

**Table 2:** Stability of expression of the 20 candidate reference genes, as calculated by BestKeeper

Ranking	***H. robusta*** -leaves		***H. robusta*** -young stems		***H. robusta*** -leaves & young stems		Different tissues		4 °C treatment		MeJA treatment		PEG6000 treatment		All samples	
	gene	CV ± SD	gene	CV ± SD	gene	CV ± SD	gene	CV ± SD	gene	CV ± SD	gene	CV ± SD	gene	CV ± SD	gene	CV ± SD
1	*UBC17*	1.16 ± 0.23	*HIS1*	1.40 ± 0.31	*UBC17*	1.73 ± 0.34	*HIS6*	5.41 ± 1.38	*18S*	0.73 ± 0.16	*60S-18*	0.67 ± 0.14	*PPIA26*	3.89 ± 0.87	*MUB1*	3.95 ± 0.87
2	*18S*	1.62 ± 0.36	*UBC17*	1.70 ± 0.33	*18S*	2.12 ± 0.47	*MUB1*	5.48 ± 1.16	*60S-18*	0.83 ± 0.18	*EF1*	0.83 ± 0.14	*60S-18*	4.48 ± 1.02	*PPIA95*	4.16 ± 0.96
3	*HIS6*	2.05 ± 0.48	*TUB-β*	1.84 ± 0.44	*MUB1*	2.54 ± 0.56	*PPIA95*	6.24 ± 1.36	*PPIA95*	0.90 ± 0.21	*HIS1*	0.85 ± 0.19	*PPIA95*	5.10 ± 1.24	*18S*	4.58 ± 1.02
4	*UBC5B*	2.06 ± 0.37	*18S*	1.87 ± 0.41	*HIS6*	2.69 ± 0.62	*SAMDC*	6.42 ± 1.34	*EF1*	1.13 ± 0.21	*TUB-β*	0.87 ± 0.20	*ACT7*	5.18 ± 1.18	*PGK*	4.90 ± 0.99
5	*PP2C59*	2.31 ± 0.45	*PGK*	2.05 ± 0.40	*HIS1*	3.09 ± 0.70	*60S-18*	6.57 ± 1.34	*PGK*	1.19 ± 0.24	*PPIA95*	0.88 ± 0.20	*HIS6*	5.26 ± 1.42	*PP2C59*	5.13 ± 1.02
6	*SAMDC*	2.84 ± 0.63	*ACT7*	2.25 ± 0.44	*UBC5B*	3.09 ± 0.54	*UBC5B*	7.32 ± 1.30	*TUB-α*	1.32 ± 0.27	*TIP41*	0.89 ± 0.20	*TUB-β*	5.32 ± 1.36	*TUB-α*	5.14 ± 1.09
7	*MUB1*	2.95 ± 0.65	*MUB1*	2.26 ± 0.50	*PPIA95*	3.17 ± 0.74	*EF2*	7.47 ± 1.48	*ACT7*	1.51 ± 0.33	*UBC17*	0.97 ± 0.19	*PGK*	5.43 ± 1.17	*TIP41*	5.28 ± 1.17
8	*HIS1*	3.06 ± 0.71	*TIP41*	2.46 ± 0.52	*PP2C59*	3.31 ± 0.66	*HIS1*	7.54 ± 1.77	*SAMDC*	1.58 ± 0.34	*60S-13*	0.97 ± 0.21	*SAMDC*	5.63 ± 1.32	*SAMDC*	5.33 ± 1.15
9	*ACT7*	3.12 ± 0.65	*PPIA95*	2.75 ± 0.64	*TUB-α*	3.42 ± 0.72	*PGK*	*2*7.62 ± 1.51	*EF2*	1.65 ± 0.35	*SAMDC*	1.34 ± 0.28	*PP2C57*	5.76 ± 1.24	*UBC5B*	5.36 ± 0.97
10	*TUB-α*	3.17 ± 0.68	*UBC5B*	2.81 ± 0.48	*ACT7*	3.66 ± 0.74	*18S*	7.75 ± 1.66	*TUB-β*	1.67 ± 0.43	*HIS6*	1.49 ± 0.34	*18S*	6.07 ± 1.44	*ACT7*	5.37 ± 1.14
11	*TIP41*	3.46 ± 0.78	*HIS6*	2.87 ± 0.65	*SAMDC*	3.85 ± 0.84	*PP2C59*	7.95 ± 1.59	*HIS6*	1.92 ± 0.44	*18S*	1.57 ± 0.34	*MUB1*	6.34 ± 1.43	*UBC17*	5.48 ± 1.11
12	*PPIA95*	3.61 ± 0.84	*60S-13*	3.05 ± 0.62	*PGK*	3.96 ± 0.80	*ACT7*	8.07 ± 1.68	*60S-13*	2.09 ± 0.47	*EF2*	1.64 ± 0.32	*UBC5B*	6.37 ± 1.27	*60S-18*	5.56 ± 1.19
13	*PGK*	4.16 ± 0.86	*TUB-α*	3.36 ± 0.70	*TUB-β*	3.98 ± 0.93	*TIP41*	8.08 ± 1.77	*MUB1*	2.11 ± 0.47	*ACT7*	1.68 ± 0.35	*EF2*	6.42 ± 1.43	*HIS1*	5.86 ± 1.37
14	*PP2C57*	4.35 ± 0.88	*EF2*	3.52 ± 0.65	*TIP41*	4.34 ± 0.94	*TUB-α*	8.09 ± 1.70	*PP2C57*	2.21 ± 0.44	*PP2C57*	1.84 ± 0.38	*EF1*	6.61 ± 1.32	*TUB-β*	6.09 ± 1.47
15	*60S-13*	4.45 ± 0.97	*PP2C59*	3.99 ± 0.81	*60S-18*	4.79 ± 1.00	*60S-13*	8.83 ± 2.03	*UBC5B*	2.23 ± 0.41	*UBC5B*	1.93 ± 0.35	*UBC17*	7.03 ± 1.55	*HIS6*	6.61 ± 1.59
16	*TUB-β*	4.75 ± 1.08	*EF1*	4.21 ± 0.69	*60S-13*	5.07 ± 1.07	*UBC17*	8.97 ± 1.81	*UBC17*	2.31 ± 0.47	*PGK*	2.00 ± 0.40	*PP2C59*	7.04 ± 1.48	*EF2*	6.82 ± 1.38
17	*EF2*	4.83 ± 0.97	*60S-18*	4.48 ± 0.91	*EF1*	5.21 ± 0.87	*TUB-β*	9.33 ± 2.19	*PP2C59*	2.51 ± 0.48	*TUB-α*	2.00 ± 0.42	*TUB-α*	7.13 ± 1.62	*60S-13*	7.61 ± 1.70
18	*60S-18*	4.84 ± 1.04	*SAMDC*	4.85 ± 1.04	*EF2*	5.38 ± 1.04	*EF1*	9.44 ± 1.57	*HIS1*	2.52 ± 0.59	*MUB1*	2.28 ± 0.49	*TIP41*	7.51 ± 1.75	*PP2C57*	7.77 ± 1.66
19	*EF1*	6.03 ± 1.04	*PP2C57*	5.49 ± 1.30	*PP2C57*	8.84 ± 1.94	*PP2C57*	12.30 ± 2.71	*TIP41*	3.11 ± 0.71	*PP2C59*	3.27 ± 0.64	*HIS1*	7.70 ± 1.97	*EF1*	8.11 ± 1.43
20	*PPIA26*	8.36 ± 2.02	*PPIA26*	5.99 ± 1.67	*PPIA26*	10.94 ± 2.84	*PPIA26*	15.21 ± 3.73	*PPIA26*	4.58 ± 1.03	*PPIA26*	4.46 ± 1.04	*60S-13*	10.75 ± 2.70	*PPIA26*	9.83 ± 2.37

### RankAggreg analysis

In this study, three algorithms were used to analyze the expression stability of 20 candidate reference genes. The gene ranking tables generated by them are different because of their different algorithms. RankAggreg is an algorithm designed to aggregate large ranking lists. It performs aggregation of ordered lists based on the rankings via the Cross-Entropy Monte Carlo algorithm or a Genetic Algorithm [34]. To provide a consensus ranking, we used RankAggreg to calculate the overall gene ranking for each experimental condition, as shown in Table 3. The consensus for the top two genes in *H. robusta* treatment on leaves and under MeJA treatment was consistent with the results of geNorm analysis. *HIS1* ranked first for young stem tissue under *H. robusta* treatment. The first-placed genes were *60S-18* and *HIS6* under 4 °C treatment and PEG6000 treatment, respectively. *TUB-α* was the most stable gene in different tissues and all samples. The expression of *PPIA*26 was the most unstable under all experimental conditions except PEG6000 treatment.

**Table 3:** Stability of expression of the 20 candidate reference genes, as calculated by RankAggreg

Ranking	***H. robusta*** -leaves	***H. robusta*** -young stems	***H. robusta*** -leaves & young stems	Different tissues	4 °C treatment	MeJA treatment	PEG6000 treatment	All samples
1	*PP2C59*	*HIS1*	*UBC17*	*TUB-α*	*60S-18*	*UBC17*	*HIS6*	*TUB-α*
2	*UBC5B*	*ACT7*	*UBC5B*	*PPIA95*	*TUB-α*	*PPIA95*	*MUB1*	*PGK*
3	*HIS6*	*TIP41*	*ACT7*	*TIP41*	*PPIA95*	*EF1*	*PP2C57*	*UBC5B*
4	*UBC17*	*PGK*	*TUB-α*	*EF2*	*ACT7*	*HIS1*	*UBC5B*	*UBC17*
5	*HIS1*	*PPIA95*	*HIS1*	*PP2C59*	*PGK*	*TIP41*	*EF1*	*TIP41*
6	*ACT7*	*UBC5B*	*PPIA95*	*UBC5B*	*PP2C57*	*ACT7*	*PGK*	*PP2C59*
7	*TUB-α*	*MUB1*	*HIS6*	*HIS1*	*UBC17*	*HIS6*	*EF2*	*ACT7*
8	*PPIA95*	*EF2*	*PP2C59*	*ACT7*	*EF2*	*EF2*	*SAMDC*	*PPIA95*
9	*PGK*	*UBC17*	*PGK*	*EF1*	*SAMDC*	*UBC5B*	*ACT7*	*HIS1*
10	*EF2*	*TUB-β*	*TIP41*	*60S-18*	*UBC5B*	*PP2C57*	*60S-18*	*EF2*
11	*TIP41*	*TUB-α*	*MUB1*	*SAMDC*	*60S-13*	*PGK*	*PPIA95*	*SAMDC*
12	*MUB1*	*MUB1*	*EF2*	*UBC17*	*HIS1*	*TUB-β*	*PPIA26*	*60S-18*
13	*PP2C57*	*60S-13*	*60S-13*	*MUB1*	*PP2C59*	*60S-18*	*UBC17*	*MUB1*
14	*SAMDC*	*EF1*	*60S-18*	*60S-13*	*18S*	*TUB-α*	*PP2C59*	*TUB-β*
15	*60S-13*	*PP2C59*	*SAMDC*	*TUB-β*	*TUB-β*	*60S-13*	*18S*	*HIS6*
16	*18S*	*18S*	*EF1*	*PGK*	*TIP41*	*MUB1*	*TUB-α*	*18S*
17	*EF1*	*HIS6*	*TUB-β*	*18S*	*EF1*	*18S*	*TIP41*	*60S-13*
18	*60S-18*	*60S-18*	*18S*	*PP2C57*	*HIS6*	*SAMDC*	*TUB-β*	*EF1*
19	*TUB-β*	*PP2C57*	*PP2C57*	*HIS6*	*MUB1*	*PP2C59*	*HIS1*	*PP2C57*
20	*PPIA26*	*PPIA26*	*PPIA26*	*PPIA26*	*PPIA26*	*PPIA26*	*60S-13*	*PPIA26*

### Validation of reference genes

**Fig. 5:**
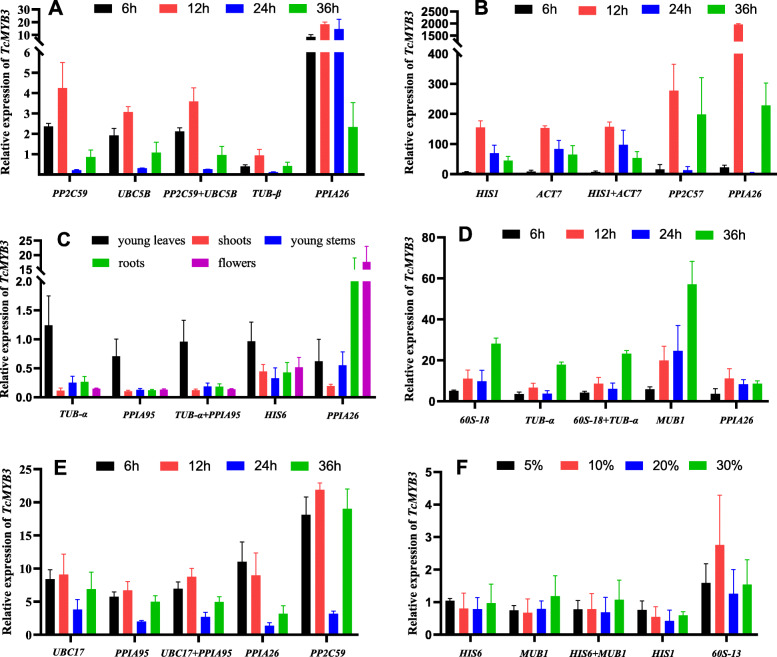
Relative expression of *TcMYB3* using the selected reference genes. The results were normalized using the selected stable reference genes (alone or in combination) and the unstable genes in sample sets across treatment with **a** *H. robusta* treatment in leaves, **b** *H. robusta* treatment in young stems, **c** different tissues, **d** 4 °C treatment, **e** MeJA treatment, **f** PEG6000 treatment. The bars indicate the standard error (±SE) evaluated from three biological replicates

In order to verify the expression stability of the selected reference genes by the software, expression of the *TcMYB3* gene was quantified using either the two most stable genes, alone and in combination, or the two most unstable genes in the consensus ranking. Under *H. robusta* treatment, the relative expression of *TcMYB3* in leaves and young stems reached a peak at 12 h when the most stable genes and their combinations were used for standardized. But the relative expression of *TcMYB3* was abnormally increased when standardized with the most unstable genes (Fig. 5a, b). As shown in Fig. 5c, when using the most stable genes as the reference genes, the expression level of *TcMYB3* increased 1–1.5 times in young leaves compared with the expression level in mature leaves and the expression of other tissues (shoots, young stems, roots, and flowers) was down-regulated and the depression multiple was basically the same. But the expression level of *TcMYB3* in the flower was the highest using the most unstable gene (*PPIA26*) as the reference gene. In addition, the expression level and trends were very similar when the most stable two reference genes and their combination were used for relative quantification under other stresses, including 4 °C treatment (Fig. 5d), MeJA treatment (Fig. 5e), and PEG6000 treatment (Fig. 5f). Whereas neither the expression level nor trend was consistent when the two most unstable internal reference genes were used for relative quantification. It is evident that the use of unstable references for gene expression analysis in *T. ciliata* can result in biased results.

## Discussion

It is ideal that reference genes are stably expressed under all experimental conditions and show stable expression levels across various tissues and growth stages of the organism, but such genes are almost non-existent [35]. More and more studies are showing that the genes that are stably expressed in different species and under different conditions change [36–39]. Selection of the most suitable reference gene for specific conditions using RT-PCR is therefore very important. This study was dedicated to discovering the best reference genes for gene expression analysis in *T. ciliata* under different conditions*.* There were 20 candidate genes from the *T. ciliata* transcriptome database screened and analyzed by RT-qPCR. It was found that the best reference genes were not consistent across different conditions. For examples, *PP2C59* and *UBC5B* were most suitable for leaves under *H. robusta* treatment, whereas *HIS1* and *ACT7* were more optimal for young stems under *H. robusta* treatment, *TUB-α* and *PPIA*95 for comparing different tissues, and *60S-18* and *TUB-α* for leaves under 4 °C treatment.

In this study, we used four methods, geNorm, NormFinder, BestKeeper, and RankAggreg, to evaluate the expression stability of 20 candidate genes. The first three algorithms were used to evaluate the expression stability of candidate genes. Our results demonstrated that reference values and calculation methods used by the three algorithms were very different [40]. NormFinder calculates stability values based on intra- and inter-group differences [25], while geNorm compares a reference gene with all genes in a given sample to evaluate the best reference gene [24]. In BestKeeper, CV and SD values determine the ranking of stability of candidate genes [41]. Due to the difference of the algorithms among the three software packages, they generated different rankings for the same set of experimental data, although the results of analysis with geNorm and NormFinder had few variations in this study. For example, *18S* was the best reference gene to use across different tissue conditions according to geNorm and NormFinder, whereas the best reference gene was *HIS6* identified by BestKeeper analysis. For young stem tissue exposed to *H. robusta* stress, *ACT7* and *UBC5B* were put forward by geNorm and NormFinder, but ranked low in BestKeeper results. In order to consolidate the results from the three algorithms, RankAggreg was used for overall ranking [34]. Many researchers use ReFinder to calculate the final ranking [42–44]. ReFinder assigns an appropriate weight to each gene and calculates the geometric mean of its weights to give the final ranking [45]. RankAggreg uses a cross-entropy Monte Carlo algorithm or genetic algorithm to produce aggregated ordered lists based on rankings [34]. Both tools play very important roles in the consolidation of the screening results for internal reference genes from other softwares.

Other researchers have studied the best reference genes for plants under pest stress, and *STP4* was found to be the best reference gene for use in *Brassica juncea* under biotic stress caused by aphid infestation [46]. *ABCT* and *FBOX* were found to be the most stable in soybean under soybean aphid (SBA) stress; *TUB4* and *TUA4* were stable under two-spotted spider mite (TSSM) stress [47]. Miranda indicated that both *GmELF1A* and *GmTUA5* were stable reference genes for normalization of expression data obtained from soybean roots infected with *Meloidogyne incognita*, and *GmCYP2* and *GmELF1A* were the best reference genes in soybean leaves infested by *Anticarsia gemmatalis* [48]. Under *H. robusta* stress, the reference genes that performed best in leaves and young stem tissues were different in our study. *PP2C59* and *UBC5B* showed high stability of expression in leaves, while only *PP2C59* ranked high for young stems. Once again, the appropriate reference genes for different species under different conditions and in different tissues vary. Hence, it is necessary to identify the best reference genes for specific conditions via RT-qPCR. Protein phosphatase can reverse the phosphorylation of protein kinases, thereby dynamically controlling protein phosphorylation and protein phosphatase 2Cs (*PP2Cs*) is the most abundant type of phosphatase in plants [49]. Although it not often has been used as a candidate internal reference gene, it is stable in our study under pest stress and in different tissues for GA treatment of *Santalum album* [50]. Therefore, when screening reference genes in other species, *PP2Cs* can be considered as a candidate reference gene.

Under most experimental conditions in this study (all except for PEG6000 treatment), the reference gene with the worst performance was *PPIA*26, which was the best reference gene recommended by BestKeeper for use under PEG6000 stress. Another gene in this family, *PPIA*95, ranked first in geNorm analysis for both 4 °C cold stress and MeJA treatment. For leaves and young stems under *H. robusta* treatment and MeJA stress, *PPIA*95 ranked third in NormFinder analysis. In BestKeeper analysis, *PPIA*95 ranked third under 4 °C cold stress and PEG6000-induced drought stress, and among all samples it ranked second. Overall, the *PPIA* gene family is a promising reference gene set in *T. ciliata*. The *PPIA* gene family encodes proteins with functions in immune responses, as well as resistance to cancer, autoimmune diseases, protozoan, and viral infections [51]. In plants, genes of the *PPIA* family are rarely used as internal reference genes, but they are abundantly expressed in the *T. ciliata* transcriptome data and the expression level in each sample is very similar, which is the main reason for choosing them. As reference genes, they are also stably expressed in animals. For example, in different heart and disease conditions, *PPIA* is recognized by ReFinder as the best reference gene in different skeletal muscles of mice, and it ranked first for human endometrial cancer [52, 53]. *PPIB* is believed as the optimal reference gene in analyzing the blood of Machado-Joseph disease (MJD) patients [54].

## Conclusion

This study is the first report about screening and verification of expression stability analysis of a series of reference genes under different conditions in *T. ciliata*, showing that the optimal reference genes were *TUB-α* and *PGK* across all samples; *PP2C59* and *UBC5B* in leaves and *HIS1* and *ACT7* in young stems under *H. robusta* treatment; *TUB-α* and *PPIA95* in different tissues; *60S-18* and *TUB-α* under 4 °C treatment; *UBC17* and *PPIA95* under MeJA treatment; *HIS1* and *MUB1* under PEG6000 treatment, respectively. We believe this research is important for accurate quantification and expression analysis of genes under different conditions in *T. ciliata*. It will play a vital role in the molecular breeding work of *T. ciliata*, such as the research on the *H. robusta-*resistant and drought-resistant varieties, as well as the research on the metabolic pathways of precious compounds in plants in the future.

## Methods

### Plant materials

Five different experiments were conducted for data collection (Table 4). Experimental samples were all collected from one-year old *T. ciliata*, grown in a greenhouse in South China Agricultural University (SCAU). For samples from different tissues, mature leaves, young leaves, flowers, shoots, and young stems were collected at 9:00 am on August 25, 2019. Before treatment with the *H. robusta*, PEG6000, 4 °C, and MeJA, all the seedlings were pre-incubated in incubator for 7 days with 16 h of light at 28 °C and 8 h of dark at 22 °C to mimic the wild environment. For *H. robusta* treatment, seedlings were exposed to herbivores, and leaves and young stems were harvested after 0, 6, 12, 24, and 36 h. After seedlings were treated with 0, 5, 10, 20, and 30% (w/v) of PEG6000 for 7 days, leaves were collected. For 4 °C treatment, seedlings were placed at 4 °C, and samples (leaves) were taken at 0, 6, 12, 24, and 36 h. Seedlings sprayed with MeJA (100 μM) were sealed in plastic bags and leaves were collected at 0, 6, 12, 24, and 36 h. Three biological replicates were taken for each sampling point, and all samples were immediately frozen in liquid nitrogen and stored at − 80 °C.

**Table 4:** Experimental details

Experimental design	Tissue	Biological repetition	Sampling points	Number of samples
Different tissues	mature leaves, young leaves, flowers, shoots, young stems, roots	3	1	18
*H. robusta* treatment	leaves, young stems	3	6	36
4 °C treatment	leaves	3	5	15
MeJA treatment	leaves	3	5	15
PEG6000 treatment	leaves	3	5	15

### RNA extraction, quality assessment, and DNA synthesis

Total RNA was extracted from all samples using a HiPure HP Plant RNA Mini Kit (Magen) with DNase treatment to remove genomic DNA. The quality of the RNA was determined with NanoDrop ND1000 (Thermo Scientific). RNA samples with absorbance ratios of A260/A280 and A260/A230 both around 2.0 were selected for further analysis. To synthesize cDNA, 0.5 μg of total RNA was used according to the manufacturer’s instructions for the HiScript II Reverse Transcript kit (Vazyme). Five-fold diluted cDNA was used for subsequent RT-qPCR experiments.

### Selection of candidate reference genes and primer design

Twenty candidate reference genes were selected from the *T. ciliata* leaf transcriptome database by reviewing previous literature: *PGK, 60S-18, 60S-13, HIS1, HIS6, PP2C57, PP2C59, UBC5B, UBC17, ACT7, SAMDC, EF1, EF2, 18S, TUB-α, TUB-β, MUB1, PPIA26, PPIA95, TIP41*. Since there is no genomic sequence data available for *T. ciliata*, we designed primers based on the sequences in the *T. ciliata* transcriptome database. Firstly, the Coding Sequence (CDS) and genomic DNA sequences (gDNA) of the candidate reference genes were amplified separately (Table S1), and then the introns and exons of the candidate reference genes were obtained by sequence alignment (NCBI-Blast), and finally primers for RT-qPCR analysis of each reference gene were designed using the web-based Primer-Blast tool from NCBI (https://www.ncbi.nlm.nih.gov/tools/primer-blast/). Apart from *60S-18, SAMDC, TUB-β* and *PPIA26,* RT-qPCR primers were designed across introns. Details of these genes and primers are shown in Table 1.

### PCR and RT-qPCR analysis

The volume of each PCR amplification reaction mix was 20 μL, containing 10 μL of Phata Max Buffer, 2 μL of five-fold diluted cDNA, 2 μL of each primer (10 μM), 0.5 μL of dNTP, 0.5 μL of Phata Max Super-Fidelity DNA Polymerase, and 5 μL ddH_2_O. The PCR reaction procedure was as follows: 95 °C for 3 min, 35 cycles of 95 °C for 15 s, 55 °C for 15 s, 72 °C for 15 s, followed by 5 min extension at 72 °C. The RT-qPCR reaction mixture consisted of 10 μL ChamQ Universal SYBR qPCR Master Mix (Vazyme), 2 μL of cDNA, 0.4 μL of each primer (10 μM), and 7.2 μL ddH_2_O to a final volume of 20 μL, and it was performed on LightCycler480 (Roche Molecular Biochemicals, Mannheim, Germany) with optical 96-well plate. To test the specificity of the RT-qPCR primers, the products of PCR were analyzed by nucleic acid electrophoresis on a 2% (w/v) gel and the melting curve was included after amplification. All samples used for RT-qPCR analysis had three biological replicates, each containing three technical replicates. In order to calculate the gene-specific PCR efficiency (E) and correlation coefficient (R^2^) of each gene, a standard curve was generated from the mixed complementary DNA (cDNA) using a fivefold dilution series.

### Analysis of stability of expression of candidate reference genes

CT values were obtained by RT-qPCR, and used to evaluate the expression levels of candidate genes in different experimental conditions and tissues. Three commonly employed algorithms, geNorm [24], NormFinder [25], and BestKeeper [26], were used to evaluate the stability of candidate reference genes in different experiments.

The package geNorm (Version3.5) screens stable reference genes by calculating the M value of the stability of each candidate gene, and the criterion is that the smaller the M value is, the higher the stability of the candidate gene is. It also calculates pairwise variations of the normalized factor after introducing a new internal reference gene, and determines the number of optimal internal reference genes based on the ratio V_n_ / V_n + 1_. If the value of V_n_ / V_n + 1_ is less than 0.15, the number of optimal internal reference genes is n. If the value of V_n_ / V_n + 1_ is greater than 0.15, the number of optimal reference genes is n + 1. NormFinder selects the most suitable internal reference gene by calculating a stability value for gene expression of the candidates. The lower the stability value is, the more stable the gene is. Using BestKeeper, the SD and CV of expression of each candidate gene can be obtained. The CV ± SD values of different genes were then compared to determine the relative stability of expression of the candidates. Finally, in order to generate an overall ranking of candidate genes from the data generated by geNorm, NormFinder, and BestKeeper, we used the RankAggreg (version 0.6.5) software package in R as previously described [14, 55–57]. RankAggreg is an algorithmic package that can combine different ranking lists. Based on the size of the rankings list, we used the Cross-Entropy Monte Carlo algorithm [34]. The rankings list previously generated by the three packages were used as input with the following parameters: the distance was calculated using Spearman’s Footrule function, with *rho* set at 0.1, the seed at 100, and the “convIn” argument at 50.

### Validation of reference genes

In order to verify the accuracy of the rankings and the stability of expression of the selected reference genes, the two most stable reference genes, alone and in combination, and the two most unstable reference genes, as recommended by RankAggreg were used to verify the relative expression of *TcMYB3* under 4 °C, MeJA, PEG6000, in different tissues, and under *H. robusta* treatment (leaves and young stems). Finally, we used the 2^-△△CT^ method to calculate the relative expression levels of the verified genes, where △CT = CT (target gene)-CT (reference gene), △△CT = △CT (treatment)-△ CT (control), 2^-△△CT^ = relative expression. Three technical replicates were performed for each biological sample [58].

## Supplementary information

**Additional file 1 Figure S1**: Amplification products of the twenty candidate reference genes and *TcMYB3.* **Figure S2**: Melting curves of candidate reference genes and *TcMYB3.*

**Additional file 2 Table S1.** Coding sequences and genomic DNA sequences of candidate reference gene.

## Data Availability

The datasets generated and/or analyzed during the current study are available in the [GeneBank] repository, [the GeneBank accession number are form MW003991 to MW004010.]

## Abbreviations

RT-qPCR: Quantitative real time PCR
*T. ciliata*: *Toona ciliata* Roem
*H. robusta*: *Hypsipyla robusta*
MeJA: Methyl jasmonate
CT: Cycle threshold
*TcMYB3*: Transcription factor MYB3
CV: Coefficient of variation
SD: Standard deviation
CDS: Coding Sequence
gDNA: Genomic DNA
cDNA: Complementary DNA
