# Supplementary material for: Selection and validation of reference genes for measuring gene expression in Toona ciliata under different experimental conditions by quantitative real-time PCR analysis
Source: BMC Plant Biol. 2020 Oct 1;20:450. doi: 10.1186/s12870-020-02670-3 (PMC7528382; doi:10.1186/s12870-020-02670-3)
Supplement: Supplementary file 1 — Additional file 1 Figure S1: Amplification products of the twenty candidate reference genes and TcMYB3. Figure S2: Melting curves of candidate reference genes and TcMYB3. [file 12870_2020_2670_MOESM1_ESM.docx]

| 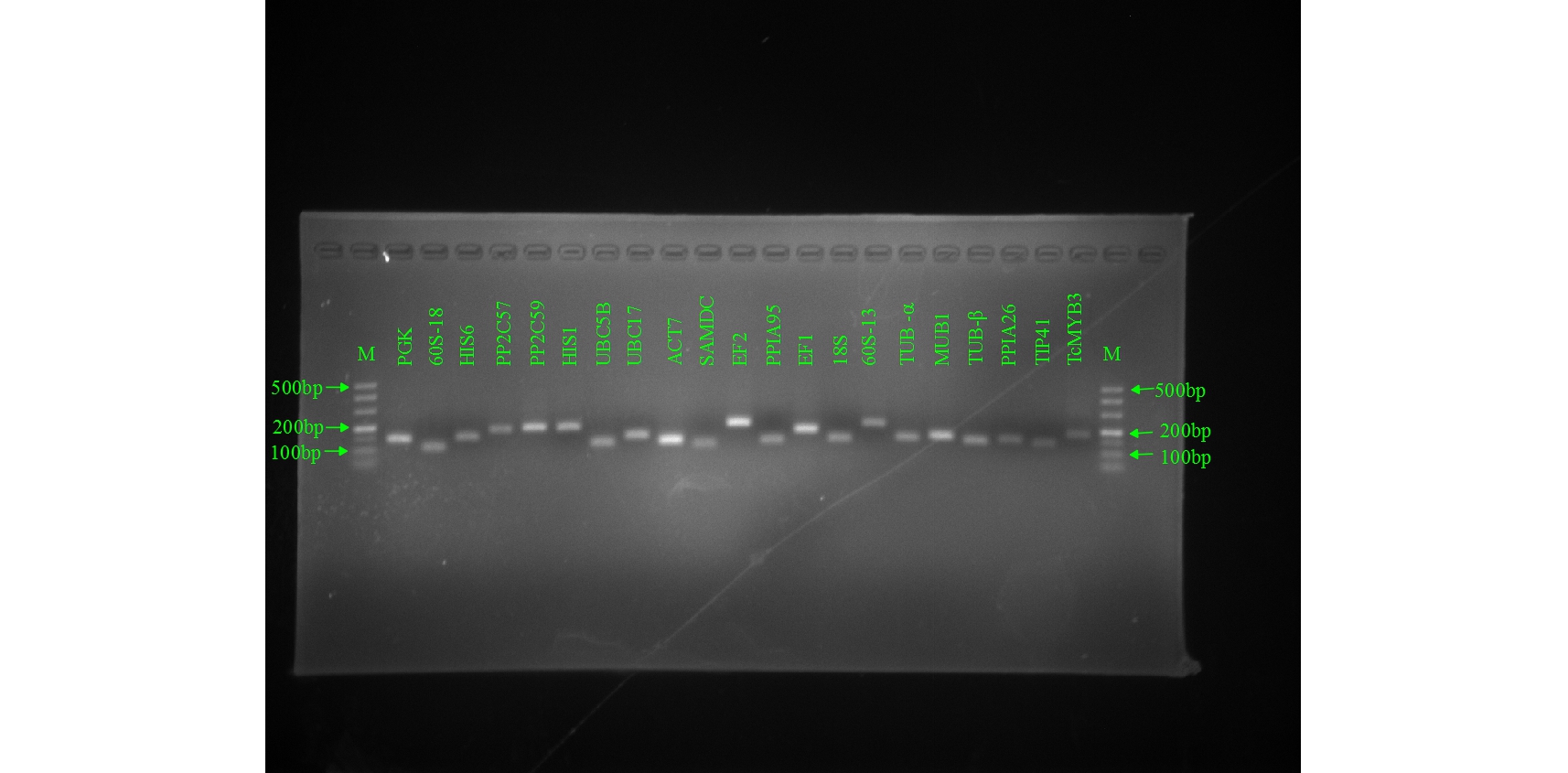 |
| --- |
| **Fig S1**: Amplification products of the twenty candidate reference genes and *TcMYB3* |

| **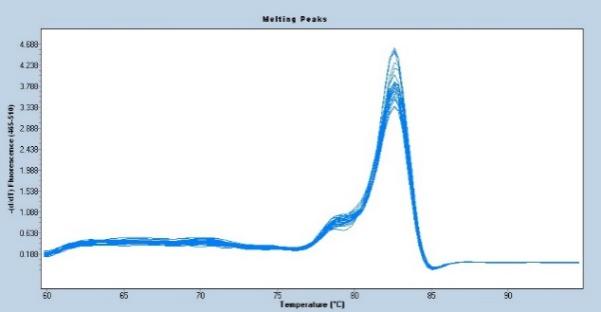**  *ACT7* | **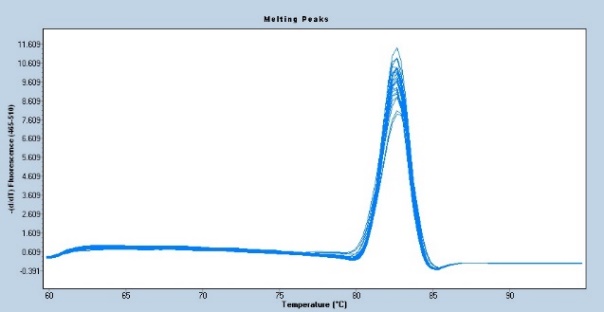**  *PGK* |
| --- | --- |
| **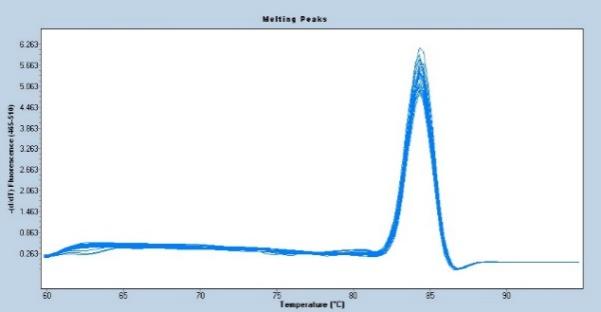**  *60S-13* | **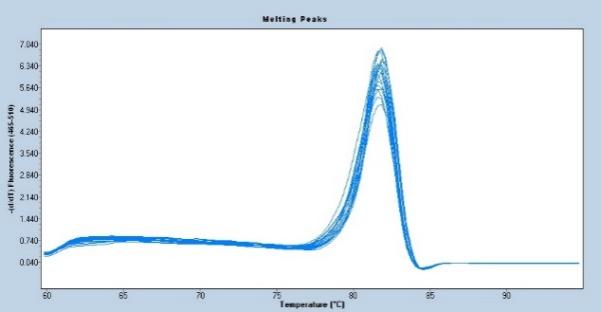**  *60S-18* |
| **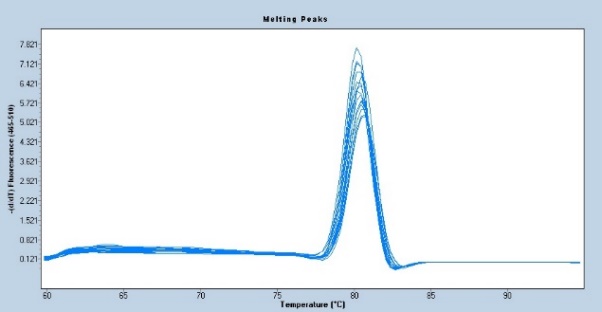**  *HIS1* | **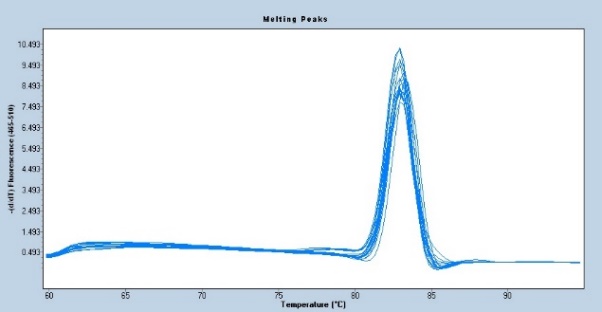**  *HIS6* |
| **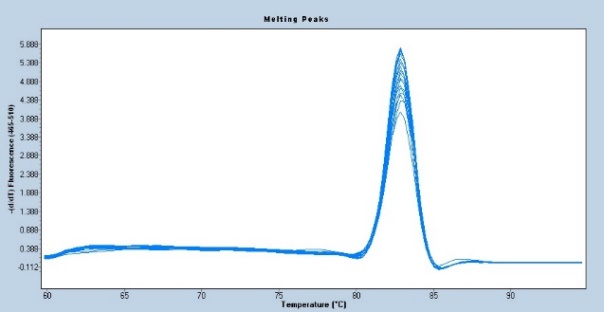**  *PP2C57* | **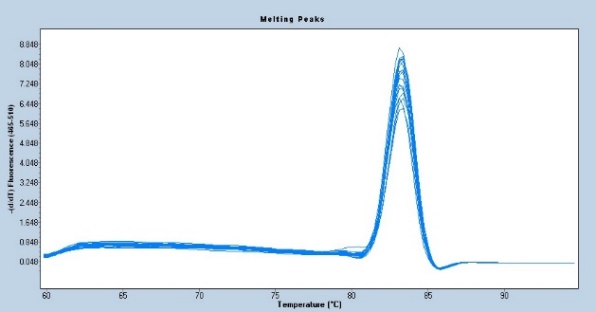**  *PP2C59* |
| **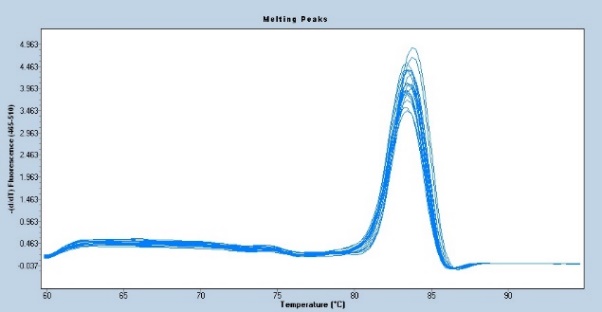**  *UBC5B* | **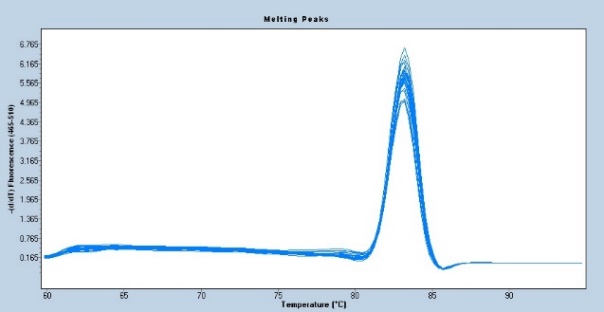**  *UBC17* |
| **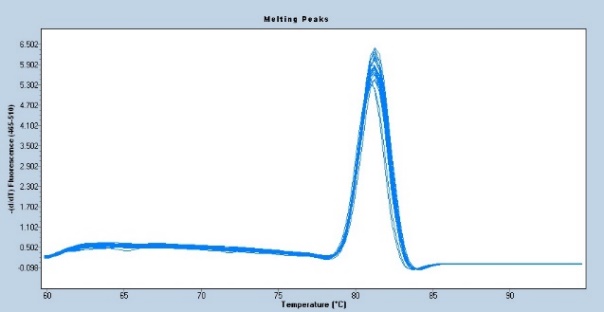**  *SAMDC* | **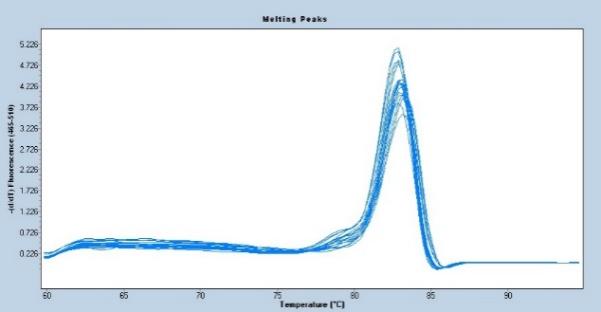**  *EF1* |
| **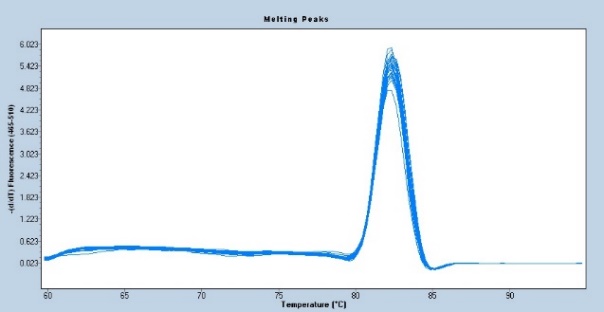**  *EF2* | **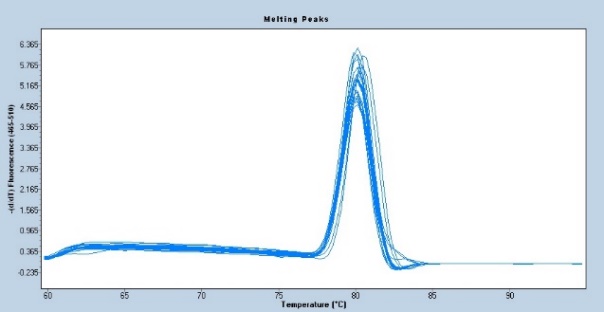**  *PPIA95* |
| **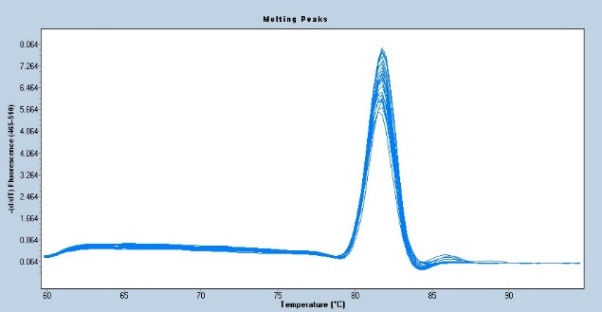**  *PPIA26* | **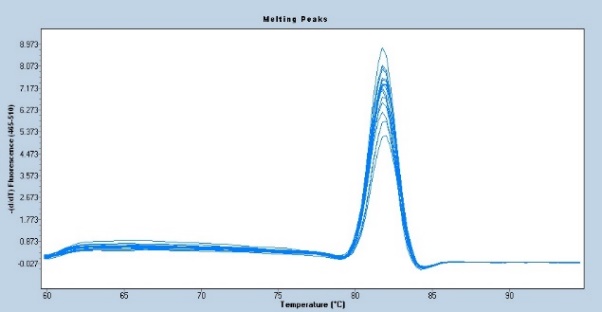**  *18S* |
| **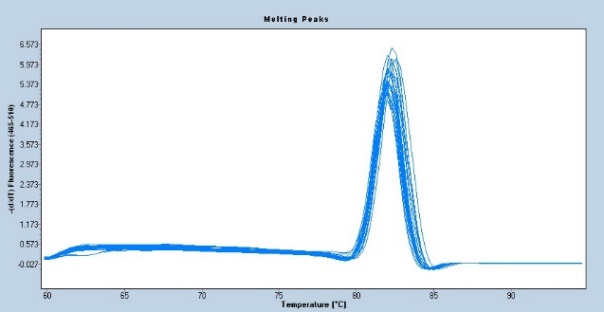**  *TUB-β* | **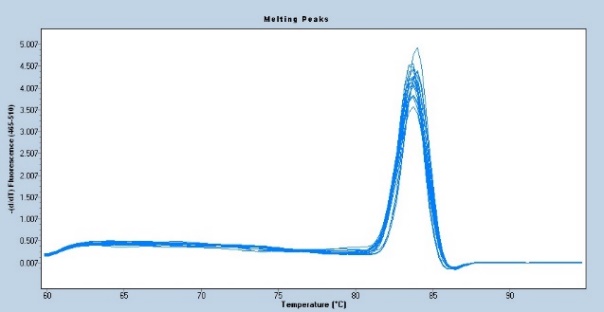**  *TUB-α* |
| **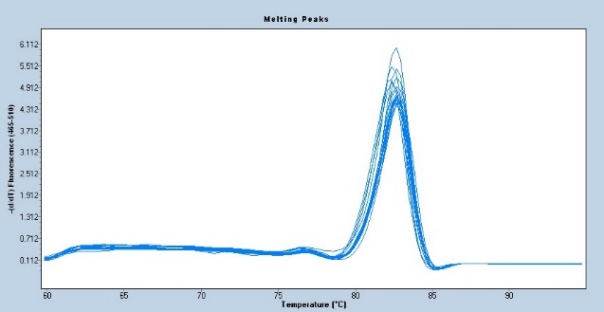**  *MUB1* | **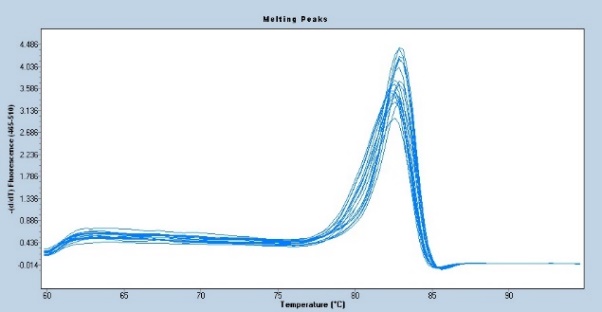**  *TIP41* |
| **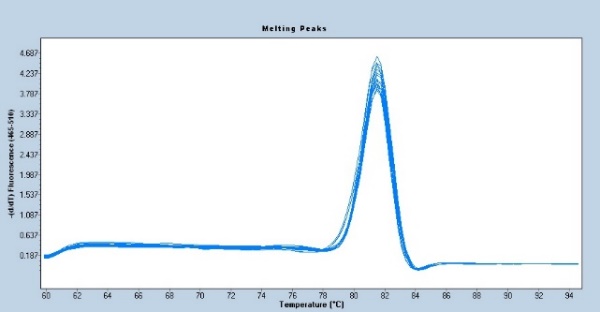**  *TcMYB3* |  |
| **Fig S2**: Melting curves of candidate reference genes and *TcMYB3* | |
